# Subtype‐specific effects of dopaminergic D2 receptor activation on synaptic trains in layer V pyramidal neurons in the mouse prefrontal cortex

**DOI:** 10.14814/phy2.13499

**Published:** 2017-11-17

**Authors:** Jonna M. Leyrer‐Jackson, Mark P. Thomas

**Affiliations:** ^1^ University of Northern Colorado School of Biological Sciences University of Northern Colorado Greeley Colorado

**Keywords:** AMPA, D2 receptor, dopamine, excitatory postsynaptic potentials, NMDA

## Abstract

In humans, prefrontal cortical areas are known to support executive functions. In mice, these functions are mediated by homologous regions in the medial prefrontal cortex (mPFC). Executive processes are critically dependent on optimal levels of dopamine (DA), but the cellular mechanisms of DA modulation are incompletely understood. Stable patterns of neuronal activity may be sensitive to frequency‐dependent changes in synaptic transmission. We characterized the effects of D2 receptor (D2R) activation on short‐term excitatory postsynaptic potential (EPSP) dynamics evoked at varying frequencies in the two subtypes of layer V pyramidal neurons in mouse mPFC. We isolated NMDA receptor and non‐NMDA receptor‐mediated components of EPSP trains evoked by stimulating fibers within layer V or layer I. All significant effects of D2 receptor activation were confined to type I (corticopontine) cells. First, we found that with layer I stimulation, D2R activation reduces the amplitude of NMDAR‐mediated EPSPs, with no effect on facilitation or depression of these responses at lower frequencies, but leading to facilitation with high frequency stimulation. Further, the non‐NMDA component also underwent synaptic depression at low frequencies. Second, with layer V stimulation, D2R activation had no effect on NMDA or non‐NMDA receptor‐mediated EPSP components. Overall, our results suggest that D2R activation may modulate memory functions by inhibiting ‘top‐down’ influences from apical tuft inputs activated at low frequencies, while promoting ‘top‐down’ influences from inputs activated at higher frequencies. These data provide further insight into mechanisms of dopamine's modulation of executive functions.

## Introduction

In humans, prefrontal cortical (PFC) areas are known to support goal‐directed behaviors, mediating a variety of functions that render behavior more flexible in the face of changing environmental demands. It has been hypothesized that the function of medial regions of the PFC is to learn associations between contextual events, and corresponding emotional responses, i.e., action‐outcome associations (Euston et al. 2012). In mice, these functions are mediated by homologous regions in the medial prefrontal cortex (mPFC) (Heidbreder and Groenewegen 2003; Seamans et al. 2008). When executive functions are disrupted, individuals suffer losses in social capabilities, which is often associated with diseases such as bipolar disorder, schizoaffective disorder, and schizophrenia. More subtle disturbances in these executive functions may cause attention deficit disorders and anxiety disorders (e.g., post‐traumatic stress disorder).

Normal prefrontal cortical function is critically dependent on dopaminergic input from the ventral tegmental area of the midbrain (Goldman‐Rakic 1995). Dopamine, acting on D1‐like and D2‐like receptors in the PFC, has been implicated in a wide variety of prefrontal functions, including updating working memory (Sawaguchi and Goldman‐Rakic 1991) and context representations (D'Ardenne et al. 2012), as well as rewarding appetitive behaviors (Arias‐Carrión and Pŏppel 2007). The major neocortical output cells located in layer V comprise two subtypes: subcortically projecting (type I) and contralaterally projecting or commissural (type II) (Molnár and Cheung 2006). It has been proposed that type I and type II layer V pyramidal neurons of the mPFC exhibit different surface expression of dopamine receptors. For example, Gee et al. (2012) have suggested that type I cells express both D1 and D2‐type receptors, while type II cells only express D1‐type receptors. Further, they hypothesize that D2‐receptors, located only on type I cells, may play a critical role in enhancing outputs to subcortical brain regions. This differential expression pattern of dopaminergic receptors on layer V pyramidal subtypes suggests that dopamine has the ability to differentially regulate these pyramidal cell subtypes. However, it is of note that both receptor types localize on GABAergic interneurons and on presynaptic excitatory glutamatergic terminals (Sesack et al. 1995; Wedzony et al. 2001).

In layer V neocortical pyramidal neurons, feedback (top‐down or contextual) information is received via apical tuft synapses (in layer I), while feedforward (bottom‐up or environmental) information is delivered to synapses on the apical trunk (Larkum et al. 2009). Further, local processing occurs between layer V neurons via synapses on basal dendrites located predominantly in layer V. When synaptic inputs to the apical tufts are activated synchronously with postsynaptic action potentials (i.e., when feedback and feedforward signals coincide), layer V pyramidal neurons act as coincidence detectors, firing in a high frequency bursting pattern, which may lead to synaptic plasticity locally and in target neurons (Larkum et al. 2009). It is conceivable that this high frequency bursting also serves as an attentional component, indicating relevant environmental information. Considered in the context of a circuit with recurrent connections (especially prominent in the mPFC), here we explore phenomena relevant to the hypothesis that layer‐specific, frequency‐dependent short‐term synaptic dynamics and their modulation by dopamine play a significant role in generating persistent activity observed in prefrontal cortical networks during memory‐related tasks.

Excitatory postsynaptic potentials (EPSPs) are characterized by two components, a non‐NMDA receptor (primarily AMPA receptor)‐mediated component that confers fast synaptic transmission, and an NMDA receptor (NMDAR)‐mediated component necessary for synaptic plasticity. Previous work characterizing dopaminergic modulation of excitatory transmission has focused on synaptic responses evoked at low frequencies. In this study, we studied the effects of D2 receptor activation on isolated NMDAR and non‐NMDAR‐mediated components of EPSPs evoked over a range of frequencies that mimic high frequency bursting, in layer V pyramidal cell subtypes. We also examined D2 receptor effects on EPSP trains evoked by stimulation of synapses in layer I (reflecting feedback activity) versus EPSP trains evoked by layer V stimulation (reflecting local connections between layer V output neurons). The intent of this study was to describe the overall effects of quinpirole on non‐NMDA and NMDA receptor‐mediated, frequency‐dependent synaptic dynamics in the two major dendritic compartments of mPFC layer V pyramids, without addressing subcellular mechanisms of D2 receptor modulation. Some of these results have been presented previously in abstract form (Leyrer‐Jackson and Thomas 2016).

## Materials and Methods

### Stereotaxic surgery

Young (28–42‐day old) mice, of both sex, were anesthetized with a combination of isoflurane and oxygen. Once under full anesthesia, the mouse was transferred to a stereotaxic frame (Stoelting 51500U, Ultra‐Precise, Wood Dale, IL), and a nose cone was placed for continuous administration of anesthesia during the surgery. A burr hole was made at coordinates ML: +0.35 mm, DV: −2.3 to −1.3 mm and +1.8 mm (for contralateral injections) and ML: −1.1, DV: −5.2 mm and RC: −3.5 mm (for pontine injections) from bregma. Coordinates were determined based on previous literature (Gee et al. 2012; Lee et al. 2014) as well as the Paxinos Brain Atlas. Lumafluor (Naples, FL) 1X green or 1X red retrobeads were injected with either a 1 *μ*L or 5 *μ*L neuros syringe (Hamilton Company, Reno, NV) at the coordinates above at volumes of 600–800 nL. Following injection, the incision site was sutured with 4‐6 stitches (Roboz RS‐7985‐12 needles; Roboz SUT‐15‐2 sutures). Mice were then subcutaneously injected with Rimadyl (Carprofen; Pfizer Pharmaceuticals, Brooklyn, NY) for pain and individually housed for 2–7 days before tissue slices were made.

### Tissue preparation

Tissue slices were prepared from 30 to 52‐day old bead‐injected mice (C57 BL/6 strain, UNC breeding colony). Animals were anesthetized with carbon dioxide and rapidly decapitated following procedures outlined in a UNC Institutional Animal Care and Use Committee approved protocol in accordance with NIH guidelines. Brains were rapidly removed and immersed in ice‐cold carbogen (95% O_2_/5% CO_2_) saturated sucrose‐enriched artificial cerebrospinal fluid (cutting aCSF) containing (in mmol/L): sucrose, 206; NaHCO_3_, 25; dextrose, 10; KCl, 3.3; NaH_2_PO_4_, 1.23; CaCl_2_, 1.0; MgCl_2_, 4.0, osmolarity adjusted to 295 ± 5 mOsm and pH adjusted to 7.40 ± 0.03. The brains were then transferred to the cutting chamber of a vibrating tissue slicer (OTS500, Electron Microscopy Sciences, Hatfield, PA) and coronal slices of the prefrontal cortex (PFC) were prepared in ice‐cold cutting aCSF. Slices were cut 300 *μ*m thick and were taken from approximately 200 *μ*m to 1400 *μ*m caudal to the frontal pole. Slices were then placed in a holding chamber filled with recording aCSF solution containing (in mmol/L): NaCl, 120; NaHCO_3_, 25; KCl, 3.3; NaH_2_PO_4_, 1.23; CaCl_2_, 0.9; MgCl_2_, 2.0; dextrose, 10, osmolarity adjusted to 295 ± 5 mOsm and pH adjusted to 7.40 ± 0.03. The holding chamber aCSF was continuously bubbled with carbogen and incubated at 34°C for 45 min and then allowed to cool to room temperature before slice recording. Prior to experiments, slices were transferred to a recording chamber where they were perfused continuously at a flow rate of 1–2 mls/min with filtered, carbogen‐saturated recording aCSF solution.

Throughout recordings, the recording chamber was held at 32 ± 1°C with a temperature controller equipped with a chamber heater and an in‐line heater (TC‐344B, Warner Instruments, Hamden CT). In experiments isolating non‐NMDAR‐mediated EPSPs, the recording aCSF contained 50 *μ*mol/L aminophosphonovalerate (D‐APV; an NMDA receptor antagonist). In experiments isolating NMDAR mediated EPSPs, the recording aCSF contained 20 *μ*mol/L 6,7dinitroquinoxaline‐2,3‐dione (DNQX; a non‐NMDA (AMPA) receptor antagonist) and recording buffer MgCl_2_ concentration was reduced to 0.25 mmol/L to facilitate NMDAR activation at ‐65 mV.

### Electrophysiology

Layer V pyramidal neurons of the infralimbic, prelimbic and anterior cingulate cortices were visually identified using infrared DIC microscopy at 400x magnification with an Olympus BX51WI microscope (Tokyo, Japan). Fluorescence was visualized using light emitted from an X‐Cite LED (Excelitas, Waltham, MA). Whole cell recordings were made from the soma of fluorescent layer V pyramidal neurons after establishing a Giga‐ohm seal (resistance range: 1–10 Gohm). Only cells that exhibited a thin (i.e., amplitude at half‐width less than 2 msec), overshooting action potential, as well as continuous spiking throughout a depolarizing current injection were used in this study. Access resistance (R_A_) was compensated throughout experiments, and cells were excluded from analysis if uncompensated R_A_ exceeded 20MΩ. Liquid junction potentials (estimated at approximately −6 mV for K^+^ gluconate internal solution) were not compensated in adjusting Vm for synaptic recordings. Amplifier bridge balance was utilized and monitored throughout current injections. Recording pipettes (4–6MΩ tip resistance), produced from thin‐wall glass capillary tubes (1.5 *μ*m OD, 1.12 *μ*m ID, World Precision Instruments, Sarasota, FL), were filled with an intracellular solution containing (in mmol/L): potassium gluconate, 135; KCl, 10; EGTA, 1.0; HEPES, 10; MgATP, 2; TrisGTP, 0.38, osmolarity adjusted to 285 ± 5 mOsm and pH adjusted to 7.30 ± 0.01. Additional glass micropipettes, filled with 3mol/L NaCl, were placed within either layer V or layer I of mPFC and used as stimulating pipettes to activate fibers located within that layer.

In addition to retrograde labeling, type I and II layer V pyramidal cells were also identified based on the presence of a prominent “sag” in response to a 150 pA hyperpolarizing current (type I: minimal 12% depolarization from peak of hyperpolarization, indicating the strong presence of the hyperpolarization‐activated cation current), and by initial firing of doublets (only observed in type I cells); both of these criteria have been used in previous studies (Dembrow et al. 2010; Gee et al. 2012; Lee et al. 2014; Spindle and Thomas 2014). For all analyses, type I and type II subtypes were categorized and compared between experimental groups. The responses were digitized at 10 kHz and saved on disk using a Digidata 1322A interface (Axon instruments) and pCLAMP version 8.1 software (Clampex program, Axon Instruments). Data were analyzed off‐line in Clampfit (Axon Instruments).

### Statistical analyses

All values are presented as mean ± SEM (standard error of the mean). All cells received every stimulus frequency (10 –50 Hz) and the D2R agonist. We performed an ANOVA on all experimental data except for comparisons between Rm and resting membrane potential (where a Student's *t* test was utilized). We used a two‐way repeated measures ANOVA to analyze the effects of drug and frequency of stimulation. The statistical model also included mouse and slice as random variables.

### Experimental protocols

We began each experiment by establishing a “stimulus current/evoked response” curve where stimulus intensity was increased while measuring the evoked EPSP amplitude. The stimulus current was adjusted to establish an unsaturated response near the midrange of this curve. The position of the stimulating pipette was located at a distance from the recorded cell to establish a baseline response ranging from 7 to 10 mV and 2 to 5 mV (for non‐NMDAR and NMDAR EPSPs, respectively). These amplitudes were chosen to avoid cell spiking during the pulse trains, where summation was often observed at higher stimulus train frequencies. EPSPs were evoked in current clamp mode using an 8‐pulse stimulus train, at varying frequencies (10–50 Hz). This protocol was repeated five times with a 10 sec intertrain interval, and the five responses were averaged. All experiments were conducted in the presence of the GABA_A_ antagonist, gabazine (10 *μ*mol/L), to isolate glutamatergic responses. For non‐NMDAR‐mediated EPSP experiments, cells were manually held at −80 mV throughout the experiment. Pulse trains were applied in control (APV‐containing) aCSF, and again immediately following a 5‐min application of the D2 agonist, Quinpirole (20 *μ*mol/L). NMDAR‐mediated EPSPs were evoked using the same current clamp protocol as for non‐NMDAR‐mediated EPSP experiments. During NMDAR EPSP experiments, cells were manually held at −65 mV throughout the experiment. Pulse trains were applied in control (low magnesium and DNQX‐containing) aCSF, and again immediately following a 5‐min application of the D2 agonist quinpirole (20 *μ*mol/L) (low magnesium, DNQX and Quinpirole‐containing). For antagonist experiments, sulpiride (10 *μ*mol/L) was included in the bath during control recordings.

EPSP amplitudes were measured in millivolts (mV), from the membrane potential directly before the stimulus was applied. The initial EPSP amplitude was measured from resting baseline, whereas subsequent EPSP amplitudes were measured from the minimal amplitude directly before the EPSP was evoked (Fig. S1A). Maximal peak amplitude was measured from baseline (before the train was delivered) to the peak amplitude reached during the train of EPSPs; the time to reach this peak amplitude within the train (time‐to‐peak) was also measured (Fig. S1B).

### Drugs

The NMDA antagonist, D‐APV, the dopamine D2‐like receptor agonist, quinpirole, the GABA_a_ antagonist, gabazine, and the dopamine D2‐like antagonist, sulpiride, were purchased from Tocris Biosciences (Bristol, UK). The non‐NMDA antagonist, DNQX, was purchased from Alomone Labs (Jerusalem, Israel). D‐APV, quinpirole, gabazine, sulpiride and DNQX were diluted into aliquots of 50 mmol/L, 20 mmol/L, 10 mmol/L, 10 mmol/L and 20 mmol/L stocks, respectively. All drugs were stored at −80°C and diluted to working concentrations of 50 *μ*mol/L D‐APV, 20 *μ*mol/L quinpirole, 10 *μ*mol/L gabazine, 10 *μ*mol/L sulpiride and 20 *μ*mol/L DNQX; any drugs not used within 3 days of thawing were discarded.

## Results

### Type I and type II cells can be distinguished based on axonal projections and electrophysiological properties

**Figure 1:**
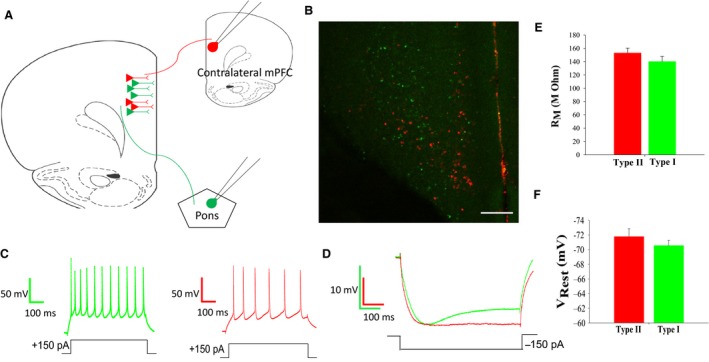
Layer V pyramidal cells of the prefrontal cortex are defined by axonal projections and intrinsic properties. (A) Schematic representation of retrograde labeling; type I cells were labeled with green retrobeads injected into the pontine of the brainstem; type II cells were labeled with red retrobeads injected into the contralateral mPFC. (B) Image showing accurately labeled type I and type II layer V pyramidal cells, with no overlap of red and green retrobeads, definitively labeling two distinct cellular populations. (scale bar = 200 *μ*m) (C) Type I cell spiking pattern showing a distinct initial spiking doublet with a 150pA current injection (green; left); Type II cell spiking pattern lacking initial spike doublet with 150pA current injection (red; right). (D) Type I cells display a large depolarizing ‘sag’ in response to a 150pA hyperpolarizing current (type I: green trace; type II: red trace). (E) Type I cells display a lower, yet nonsignificant, average R_M_ (140 ± 8.0 mΩ) than type II cells (153 ± 7.4 mΩ). (F) Type I cells display a more depolarized, yet nonsignificant, average membrane potential (‐70.6 ± 0.7 mV) than type II cells (‐71.8 ± 1.1 mV). (Student's *t* test)

Using a retrograde labeling technique, two subtypes of layer V pyramidal neurons were identified based on their axonal projection patterns. We identified two distinct subtypes of pyramidal cells that projected to either the brainstem (corticopontine/type I cells) or the commissural mPFC (commissural/type II cells), with no overlap in populations (Fig. 1B). As demonstrated in previous studies (Dembrow et al. 2010; Gee et al. 2012; Spindle and Thomas 2014), these two populations also showed differences in electrophysiological properties, where type I neurons often displayed an initial spiking doublet in response to a 150pA depolarizing current (Fig. 1C, left, green trace) and type II neurons displayed a relatively constant spiking pattern in response to the same current (Fig. 1C, right, red trace). Additionally, Type I neurons displayed a prominent depolarizing sag due to a hyperpolarization‐activated cation current in response to a 150pA hyperpolarizing current, compared to Type II neurons (Fig. 1D; type I, green; type II, red). Type I and type II neurons did not differ significantly in regards to membrane resistance (R_M_) or resting membrane potential (V_REST_) (Fig. 1E and F).

### Non‐NMDA‐receptor‐mediated EPSPs

**Figure 2:**
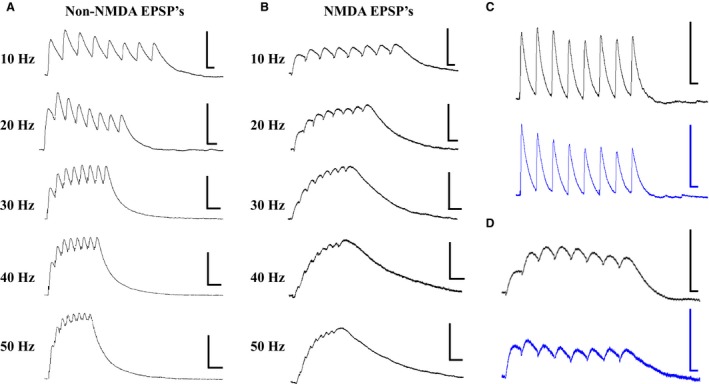
EPSP trains evoked by layer V stimulation. (A) Representative traces showing non‐NMDAR‐mediated EPSP trains evoked by layer V stimulation. Traces are shown for each stimulation frequency (10‐50 Hz from top to bottom). (B) Representative traces showing NMDAR‐mediated EPSP trains evoked by layer V stimulation. Traces are shown for each stimulation frequency (10‐50 Hz from top to bottom). (C) non‐NMDAR‐mediated EPSP trains show two types of short‐term dynamics, facilitating (EPSP2 >  EPSP1; black trace, top) and depressing (EPSP2 < EPSP1; blue trace, bottom). (D) NMDAR‐mediated EPSP trains show facilitating (EPSP2 >  EPSP1; black trace, top) and depressing (EPSP2 < EPSP1; blue trace, bottom) short‐term dynamics. All scale bars, x = 10 mV and y = 50 ms.

Non‐NMDA receptor (non‐NMDAR)‐mediated EPSP trains were measured from layer V pyramidal neurons by blocking NMDA receptor activation with 50 *μ*mol/L APV. Representative traces, for all frequencies (10–50 Hz), recorded following layer V stimulation are shown in Figure 2A. At a frequency of 10 Hz, non‐NMDAR EPSPs show two distinct amplitude profiles, which we define simply as facilitating (the second EPSP is larger than the first EPSP; EPSP2 > EPSP1; Fig. 2C, top; black trace) or depressing (the second and subsequent EPSPs are smaller than the first EPSP; EPSP2 < EPSP1; Fig. 2C, bottom; blue trace). In 23 out of 27 layer V cells (15 type I; 8 type II), layer V‐evoked non‐NMDAR EPSPs showed a depressing pattern in control solution. In the remaining four of 27 cells (2 type I, 2 type II), layer V‐evoked non‐NMDAR EPSPs were facilitating. Further, in 21 out of 22 cells (15 type I, 6 type II), layer I‐evoked non‐NMDAR EPSPs were depressing, while one cell (type II) showed a facilitating pattern. Notably, we did not see a significant difference between type I and type II cells with regard to their short‐term synaptic dynamics (layer V‐evoked non‐NMDAR EPSPs [EPSP2/EPSP1 *P* > 0.05; *n* = 27]; layer I‐evoked non‐NMDAR EPSPs [EPSP2/EPSP1; *P* > 0.05; *n* = 22]).

### NMDA‐receptor‐mediated EPSPs

NMDA receptor (NMDAR)‐mediated EPSP trains were measured from layer V pyramidal neurons by blocking non‐NMDA receptors with 20 *μ*mol/L DNQX. Representative traces, for all frequencies (10–50 Hz), recorded following layer V stimulation are shown in Figure 2B. Like non‐NMDAR EPSPs, NMDAR EPSPs also showed two distinct amplitude profiles, which we have defined as facilitating and depressing. A representative trace of an NMDAR EPSP facilitating and depressing response is shown (Fig. 2D; top (black trace) and bottom (blue trace), respectively). In 29 out of 34 layer V cells (15 type I, 14 type II), layer V‐evoked NMDAR EPSPs showed a depressing pattern in control solution. In the remaining five cells (4 type I, 1 type II), layer V‐evoked NMDAR EPSPs were facilitating. Moreover, in 19 out of 22 cells (7 type I, 12 type II), layer I‐evoked NMDAR EPSPs showed a depressing pattern, while the remaining three cells (1 type I, 2 type II) showed a facilitating pattern. Again, we did not see a significant difference between type I and type II cells with regard to their short‐term synaptic dynamics (layer V‐evoked NMDAR EPSPs [EPSP2/EPSP1; *P* > 0.05; *n* = 34]; layer I‐evoked NMDAR EPSPs [EPSP2/EPSP1; *P* > 0.05; *n* = 22]).

### Frequency‐dependent properties of EPSP trains

**Figure 3:**
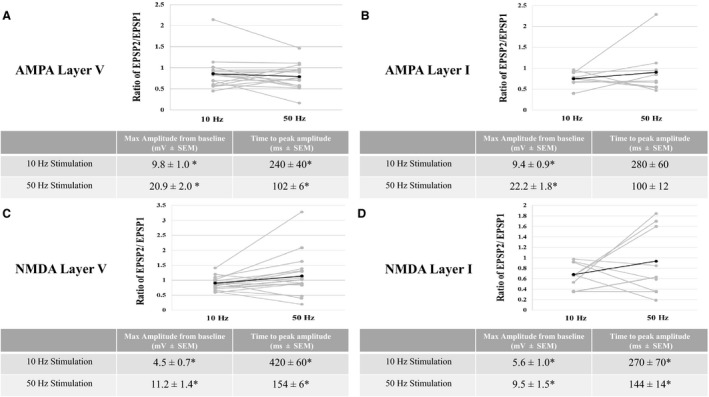
Frequency‐dependent properties of non‐NMDA and NMDA EPSPs evoked by either layer V or layer I stimulation. With layer V (A) and layer I (B) stimulation, for non‐NMDA EPSPs, the ratio of EPSP2/EPSP1 is unchanged from 10 Hz to 50 Hz (top). Addition characteristics are tabled (bottom). With layer V (C) and layer I (D) stimulation, for NMDA EPSPs, the ratio of EPSP2/EPSP1 is unchanged from 10 Hz to 50 Hz (top). Addition characteristics are tabled (bottom). For each graph, gray lines represent each individual cell and black lines represent the average of all cells. Asterisks in tables depict a significant difference (*P* < 0.05; repeated measures ANOVA) between 10 Hz and 50 Hz.

We examined the frequency‐dependent properties of both non‐NMDAR and NMDAR‐mediated EPSPs by stimulating at various frequencies between 10 Hz and 50 Hz. Non‐NMDAR‐mediated EPSPs showed similar trends in frequency‐dependent properties with layer V and layer I stimulation. For both layer I and layer V stimulation, the ratio of EPSP2/EPSP1 was not different between 10 Hz and 50 Hz (Fig. 3A&B). However, with both layer V and layer I stimulation, the maximal amplitude is higher, and the latency to peak is shorter, at 50 Hz compared to 10 Hz (Fig. 3A and B, tables). Thus, temporal summation of non‐NMDAR EPSPs is more robust at higher frequencies. However, with layer I stimulation, the latency to peak amplitude remains unchanged from 10 to 50 Hz although there is a trend for the latency to be shorter at 50 Hz (*P* = 0.06).

NMDAR‐mediated EPSPs showed similar trends in frequency‐dependent properties with layer V and layer I stimulation. For both layer I and layer V stimulation, the ratio of EPSP2/EPSP1 was not different between 10 Hz and 50 Hz (Fig. 3C and D). However, with both layer V and layer I stimulation, the maximal amplitude is higher, and the latency to peak is shorter, at 50 Hz compared to 10 Hz (Fig. 3C and D, tables). These results indicate that, regardless of layer stimulation, temporal summation of NMDAR‐mediated EPSPs is more robust at higher frequencies.

### D2 receptor effects on EPSP trains

#### Layer I‐evoked non‐NMDAR EPSPs

**Figure 4:**
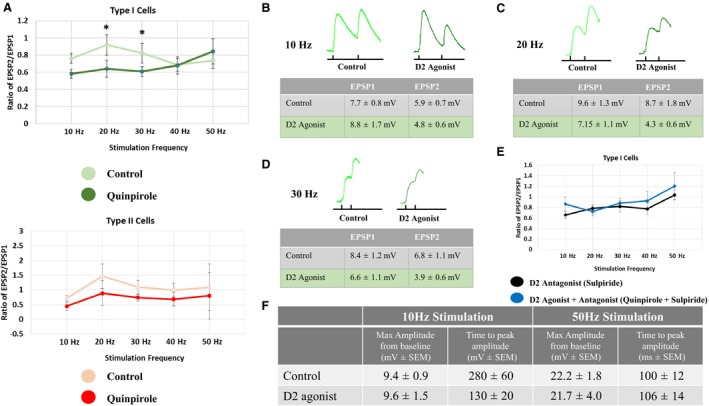
D2 receptor modulation of non‐NMDA EPSPs evoked by layer I stimulation. (A) D2 receptor activation decreases the ratio of EPSP2/EPSP1 compared to the controls significantly at 10 Hz, 20 Hz and nearly significant at 30 Hz (*P* = 0.08) in type I (top) but not type II cells (bottom). (B‐D) Representative traces highlighting EPSP1 and EPSP2 are shown for type I cells in control solution and in the presence of the D2 agonist for 10‐30 Hz. Black dashes represent stimulation time points. Amplitude of EPSP 1 and EPSP 2 are shown (tables) for stimulation frequencies of 10‐30 Hz. (E) The effects of quinpirole on the ratio of EPSP2/EPSP1, in type I cells, are blocked with the D2 antagonist, sulpiride. (F) The effects of the D2 agonist on maximum amplitude and time to peak amplitude at 10 and 50 Hz. Asterisks (*) indicate *P* < 0.05; repeated measures ANOVA.

The D2 agonist, quinpirole, had no effect on the initial EPSP amplitude in either cell type (type I cells, from 7.7 ± 0.8V to 8.8 ± 1.7 mV at 10 Hz (*P* > 0.05; *n* = 9); type II cells, from 9.9 ± 2.4 mV to 9.1 ± 2.2 mV (*P* > 0.05; *n* = 3)). The effects of quinpirole on synaptic trains were frequency‐dependent. At lower frequencies, quinpirole decreased the ratio of EPSP2/EPSP1 in type I (trending toward significance at 10 Hz, and highly significant at 20 Hz and 30 Hz), but not type II cells compared to controls (Fig. 4A). Thus, D2 receptor activation increases synaptic depression at low train frequencies only in type I cells. These effects on type I cells were blocked by the D2 antagonist, sulpiride (Fig. 4E). However, quinpirole had no effect on peak amplitude from baseline or the latency to peak amplitude at any frequency, compared with controls (data shown for 10 and 50 Hz; Fig. 4F).

#### Layer V‐evoked non‐NMDAR EPSPs

Quinpirole had no significant effects on non‐NMDAR EPSP trains evoked by layer V stimulation. In both type I and type II cells, quinpirole did not alter the initial EPSP amplitude (type I cells, from 7.9 ± 1.2V to 9.6 ± 1.2 mV at 10 Hz (*P* > 0.05; *n* = 10); type II cells, from 8.3 ± 1.4 mV to 9.4 ± 0.9 mV at 10 Hz (*P* > 0.05; *n* = 7)) or the ratio of EPSP2/EPSP1 at any frequency (data not shown). Further, quinpirole had no effect on peak amplitude from baseline, or latency to peak at any frequency compared with controls (data shown for 10 and 50 Hz; table 1).

**Table 1:** D2 receptor activation has no significant effect on frequency‐dependent properties of layer V‐evoked non‐NMDA EPSPs

	10 Hz Stimulation		50 Hz Stimulation	
	Max Amplitude from baseline (mV±SEM)	Time to peak amplitude (ms±SEM)	Max Amplitude from baseline (mV±SEM)	Time to peak amplitude (ms±SEM)
Control	9.8 ± 1.0	240 ± 40	20.9 ± 2.0	102 ± 6
D2 agonist	10.9 ± 0.8	160 ± 10	25.5 ± 2.0	104 ± 8

Compared to control solution, the D2 agonist did not significantly change the peak amplitude from baseline or the latency to peak within the stimulus train at any frequency. (repeated measures ANOVA).

#### Layer I‐evoked NMDAR EPSPs

**Figure 5:**
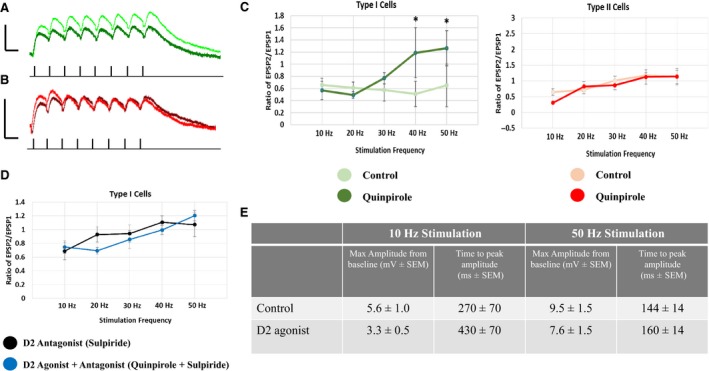
D2 receptor modulation of NMDA EPSPs evoked by layer I stimulation. (A) D2 receptor activation with quinpirole significantly decreases the amplitude of NMDA EPSPs in type I pyramidal neurons. Representative traces of type I cells, evoked at 10 Hz, are shown (control: green trace; quinpirole: dark green trace). (B) D2 receptor activation with quinpirole has no effect on the amplitude of NMDA EPSPs in type II pyramidal neurons. Representative traces of type II cells, evoked at 10 Hz, are shown (control: red trace; quinpirole: dark red trace). Black dashes represent stimulation time points; scale bars: x = 100 ms; y = 5 mV. (C) The EPSP amplitude ratio of EPSP2/EPSP1 was significantly increased at 50 Hz in type I (left) but not type II cells (right). (D) The effects of quinpirole on the ratio of EPSP2/EPSP1, in type I cells, are blocked with the D2 antagonist, sulpiride. (E) The effects of the D2 agonist on maximum amplitude and time to peak amplitude at 10 and 50 Hz. Asterisks (*) indicate *P* < 0.05; repeated measures ANOVA.

Quinpirole significantly decreased the amplitude of the first EPSP in type I cells, but not type II cells (type I cells, from 4.3 ± 1.7V to 1.4 ± 0.3 mV at 10 Hz (*P* < 0.05; *n* = 6); type II cells, 3.9 ± 1.1V to 2.0 ± 0.6 mV at 10 Hz (*P* > 0.05; *n* = 6)). Representative NMDAR EPSP traces recorded from type I and type II cells, evoked by layer I stimulation are shown in control solution (low magnesium, GZ and DNQX‐containing; Fig 5A, type I: green; Fig. 5B, type II: red) and in the presence of the D2 agonist (low magnesium, GZ, DNQX and Quinpirole; Fig. 5A; type I: dark green traces; Fig. 5B, type II: dark red traces). However, quinpirole significantly increased the ratio of EPSP2/EPSP1 in type I but not type II cells, at 40 Hz and 50 Hz (Fig. 5C). Thus, quinpirole leads to facilitation of NMDAR EPSPs in type I cells at higher frequencies. The effects of quinpirole on the initial EPSP amplitude and the ratio of EPSP2/EPSP1 in type I cells were blocked by the D2 receptor antagonist, sulpiride (data not shown and Fig. 5D, respectively). D2 receptor activation had no effect on peak amplitude from baseline or latency to peak at any frequency (data shown for 10 and 50 Hz; Fig. 5E).

#### Layer V‐evoked NMDAR EPSPs

D2 receptor activation had no effect in either cell type on initial EPSP amplitude evoked by layer V stimulation (type I cells, from 3.1 ± 0.6 mV to 2.7 ± 0.4 mV at 10 Hz (*P* > 0.05; *n* = 10); type II cells, 4.1 ± 1.4 mV to 1.0 ± 0.2 mV at 10 Hz (*P* > 0.05; *n* = 10)), or on the ratio of EPSP2/EPSP1 at any frequency (data not shown). Furthermore, D2 receptor activation also had no effect on peak amplitude from baseline or the latency to peak, at any frequency (data for 10 and 50 Hz shown; table 2).

**Table 2:** D2 receptor activation has no significant effect on frequency‐dependent properties of layer V‐evoked NMDA EPSPs

	10 Hz Stimulation		50 Hz Stimulation	
	Max Amplitude from baseline (mV±SEM)	Time to peak amplitude (ms±SEM)	Max Amplitude from baseline (mV±SEM)	Time to peak amplitude (ms±SEM)
Control	4.5 ± 0.7	420 ± 60	11.2 ± 1.4	154 ± 6
D2 agonist	4.2 ± 0.7	510 ± 60	9.3 ± 1.6	156 ± 4

Compared to control solution, the D2 agonist did not significantly change the peak amplitude from baseline or the latency to peak within the stimulus train at any frequency. (repeated measures ANOVA).

## Discussion

### Summary

In the present study, type I (corticopontine) and type II (commissural) layer V pyramidal cells were identified using fluorescent retrobeads, and the effects of D2 receptor activation on high frequency synaptic trains evoked by apical tuft (layer I) or basal dendritic (layer V) stimulation were characterized. The main results were as follows: (1) Isolated non‐NMDAR and NMDAR‐mediated EPSPs display some differences in frequency‐dependent properties, but display predominantly depressing EPSP trains. (2) D2 receptor activation modulates type I, but not type II, layer V pyramidal neurons. In type I cells, D2 receptor activation (3) increases synaptic depression of layer I‐evoked non‐NMDAR‐mediated EPSPs at lower frequencies, (4) had no effect on layer V‐evoked non‐NMDAR‐mediated EPSPs at any frequency, (5) decreases the initial EPSP amplitude and leads to facilitation at higher frequencies in layer I‐evoked NMDAR‐mediated EPSPs, and lastly (6) had no effect on layer V‐evoked NMDAR‐mediated EPSPs at any frequency.

### Comparisons with previous studies

As other laboratories have demonstrated, we have shown that layer V pyramidal cell subtypes are unambiguously distinguished based on their axonal projection patterns (Dembrow et al. 2010; Gee et al. 2012; Lee et al. 2014) using retrograde transport of fluorescent beads, since type I and type II cells are known to send axonal projections to the pontine nuclei of the brainstem and the commissural mPFC, respectively. As observed in previous studies (Dembrow et al. 2010; Spindle and Thomas 2014), we found that these subtypes differ in their intrinsic properties, where type I cells often display an initial spiking doublet and a large ‘sag’ due to a hyperpolarization‐activated inward current, and type II cells do not. The results of this study show that dopaminergic D2 receptor activation only affects type I pyramidal cells, supporting the idea that type I, but not type II, pyramidal cells express D2 receptors, which had been previously proposed by Gee et al. (2012).

To our knowledge, this is the first study to identify the effects of D2 receptor activation on frequency‐dependent synaptic properties in layer V pyramidal cells. However, frequency‐dependent synaptic properties have been studied in various cell populations. Within the nucleus accumbens and the ventral tegmental area, Chuhma et al. (2009) reported that summation (i.e., a higher peak amplitude is reached within the train) occurs much more readily when cells are stimulated at high frequencies. The same phenomenon has been shown in the ventrobasal thalamus (Castro‐Alamancos 2002a,b), as well as within CA1 pyramidal cells of the hippocampus (Davies and Collingridge 1996). Lastly, this phenomenon has also been demonstrated in layer V pyramidal cells, evoked with dendritic current injections varying in frequency (Dembrow et al. 2015). These studies, in addition to ours, demonstrate that temporal summation is enhanced at higher frequencies, and suggest that high frequency synaptic activation may facilitate persistent firing.

There is strong evidence that dopaminergic D2 receptor activation is crucial for cognitive PFC‐related functions (Arnsten et al. 1995; Druzin et al. 2000) and has been shown to improve memory performance in primates (Arnsten et al. 1995); however, the physiology behind the phenomenon remains unclear and controversial. Similar to our results of D2 receptor activation on layer I‐evoked NMDAR‐mediated EPSPs, others have reported that D2 receptor activation decreases the amplitude of layer II/III evoked EPSPs (Tseng and O'Donnell 2007) and layer V‐evoked IPSCs (Seamans et al. 2001; Trantham‐Davidson et al. 2004) in layer V pyramidal neurons of the mouse mPFC. D2 receptor activation has also been reported to suppress NMDAR‐mediated responses in CA1 pyramidal neurons of the hippocampus, evoked by stimulation of Schaffer collaterals, (Kotecha et al. 2002), in the neostriatum (Cepeda and Levine 1998) and in rat mPFC layer V pyramidal neurons, evoked by layer I and VI stimulation (Law‐Tho et al. 1994) or bath applied NMDA (Zheng et al. 1999). Further, D2 receptor activation decreases excitatory response amplitude within the nucleus accumbens of adolescent rats (Grace 2000; Benoit‐Marand and O'Donnell 2008). In combination, these studies suggest that D2 receptor activation may attenuate synaptic plasticity by depressing the amplitude of both nonisolated EPSPs as well as NMDAR‐mediated EPSPs, similar to that observed in layer I‐evoked NMDAR‐mediated EPSPs in the current study.

While modulation of single, low‐frequency EPSP properties by D2 receptor activation has been previously studied, to our knowledge minimal research has been conducted to identify changes in high‐frequency synaptic trains elicited by D2 receptor activation, including facilitation (EPSP2 > EPSP1) and depression (EPSP2 < EPSP1) at different frequencies. One study that has explored this phenomenon in nonisolated EPSC's reports similar results to those seen in layer I‐evoked NMDAR‐mediated EPSPs in the current study. Studying synaptic connections within the nucleus of the solitary tract, Kline et al. report that nonisolated EPSC's display paired‐pulse depression in control solution. With D2 receptor activation, the ratio of EPSP2/EPSP1 was enhanced from control responses (Kline et al. 2002). This study also reported that D2 receptor activation decreased the amplitude of the initial EPSC, but had minimal effect on the second evoked EPSC, similar to our results. Because D2 receptor activation decreases the initial EPSC1 amplitude, with a lesser effect on EPSC2, it has been hypothesized that D2 receptor activation may promote a decrease in presynaptic quantal release rather than a decrease in postsynaptic glutamate sensitivity (Kline et al. 2002). It is difficult to compare isolated NMDAR‐mediated EPSPs evoked in the PFC with nonisolated EPSCs of the nucleus of the solitary tract; however, our results suggest a postsynaptic locus for quinpirole's effects in the mPFC, since a presynaptic mechanism would likely have resulted in changes of non‐NMDA receptor synaptic dynamics as well as NMDAR‐mediated responses.

While many studies quantifying NMDAR‐mediated synaptic responses use low magnesium containing extracellular aCSF solutions, as in the current study, we acknowledge that these conditions could conceivably alter the short‐term synaptic dynamics of NMDAR‐mediated responses, or their modulation by quinpirole. To our knowledge, the effects of alterations in extracellular [Mg^2+^] on short‐term synaptic dynamics of NMDAR‐mediated responses have not been studied. Thus, it remains to be determined whether the frequency‐dependent effects of quinpirole would be altered under conditions of normal magnesium concentrations.

### Implications for prefrontal cortical function

The mPFC is highly interconnected with other cortical regions, where afferents target both apical and somatic regions. The current study suggests that dopamine, through D2 receptor activation, has compartmentalized effects on non‐NMDAR and NMDAR‐mediated EPSPs on layer V pyramids. First, we found that D2 receptor activation reduces the amplitude of layer I‐evoked NMDAR‐mediated EPSPs, yet had no effect on facilitation or depression of these responses at lower frequencies. For this reason, we hypothesize that dopamine, acting via D2 receptors, may play a role in limiting excessive influences of “top‐down” signals by decreasing EPSP amplitude and stabilizing low frequency evoked NMDAR‐mediated responses at apical tuft synapses on type I cells. However, with high‐frequency stimulation, D2R activation leads to facilitation of NMDAR‐mediated EPSPs, which may promote the influence of high‐frequency inputs targeting the apical tuft region. Thus, D2R activation may promote synaptic plasticity in the tufts, but only when inputs are activated at higher frequencies. Although synaptic plasticity may be enhanced at higher frequencies, the non‐NMDA component was depressed at lower frequencies with D2R activation, indicating that fast synaptic transmission is blunted at layer I synapses of type I cells. Combined, these results would amount to an effective *increase in the signal‐to‐noise ratio*, assuming that synapses activated at high frequency are carrying more pertinent information. In contrast, with layer V stimulation, D2R activation had no effect on non‐NMDAR‐mediated or NMDAR‐mediated EPSPs. This implies that D2R activation stabilizes fast synaptic transmission and plasticity at basal dendritic synapses of type I cells.

Overall, our results suggest that one role of D2R activation in modulating memory functions may thus be to inhibit influences from tuft inputs activated at low frequencies while promoting influences from tuft inputs activated at higher frequencies. Our demonstration of differential regulation of synaptic dynamics within distinct compartments of PFC layer V output neurons by D2 receptor activation may provide further insight into the mechanisms by which dopamine modulates mPFC function.

### Implications for schizophrenia

The dopaminergic system is highly dysregulated in patients with schizophrenia, contributing in part to the schizophrenic phenotype. Antipsychotics, in part, are effective by blocking dopaminergic D2‐like receptors, with a general therapeutic dose occupying 60–80% of all D2 receptors in treated patients (Seeman 2010). However, the cellular mechanisms responsible for the antipsychotic effects of D2 receptor antagonism are poorly understood. We hypothesize that during times of elevated dopamine in schizophrenic patients, abnormally enhanced D2 receptor activation leads to excessive synaptic plasticity when tuft inputs are firing at high frequencies and thus inappropriate contextual associations are made by the patient. We further hypothesize that antipsychotic drugs may in part elicit their therapeutic effects by preventing this excessive plasticity and thus preventing the false attributions regarding contextual information. However, for a more complete understanding of how dopamine regulates PFC function in the normal and pathological brain, it will be important to integrate the effects of D2 receptor activation with the effects of D1 receptor activation.

## Conflict of Interest

The authors declare that the research was conducted in the absence of any commercial or financial relationships that could be construed as a potential conflict of interest.

## Data Accessibility

## Supporting information




**Figure S**1: Analysis of EPSP characteristics. (A) Example traces of 10 Hz (top) and 50 Hz (bottom), depicting how amplitude of EPSPs was measured throughout the train. Although all EPSPs within the train were measured, for simplicity, only EPSP1, EPSP2 and EPSP8 are shown here. (B) Example traces for 10 Hz (top) and 50 Hz (bottom), are shown to display how maximal/peak amplitude was measured throughout the train.
