# Supplementary material for: Subtype‐specific effects of dopaminergic D2 receptor activation on synaptic trains in layer V pyramidal neurons in the mouse prefrontal cortex
Source: Physiol Rep. 2017 Nov 17;5(22):e13499. doi: 10.14814/phy2.13499 (PMC5704077; doi:10.14814/phy2.13499)
Supplement: Supplementary file 1 — Figure S1: Analysis of EPSP characteristics. (A) Example traces of 10 Hz (top) and 50 Hz (bottom), depicting how amplitude of EPSPs was measured throughout the train. Although all EPSPs within the train were measured, for simplicity, only EPSP1, EPSP2 and EPSP8 are shown here. (B) Example traces for 10 Hz (top) and 50 Hz (bottom), are shown to display how maximal/peak amplitude was measured throughout the train. [file PHY2-5-e13499-s001.pdf]

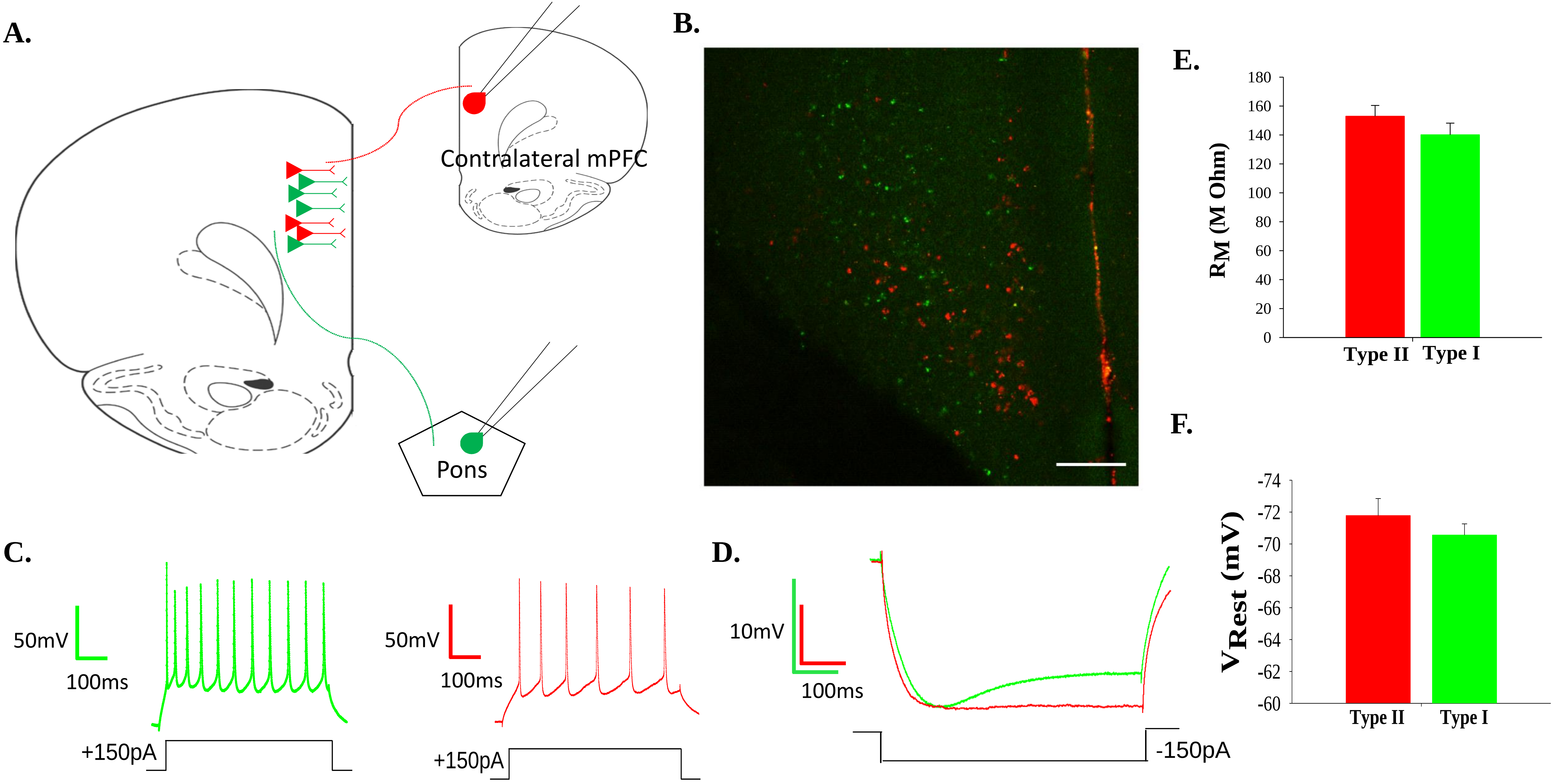
A.
Contralateral mPFC
Pons
B.
E.
R_M (M Ohm)
180
160
140
120
100
80
60
40
20
0
Type II
Type I
F.
V_{Rest} (mV)
-74
-72
-70
-68
-66
-64
-62
-60
Type II
Type I
C.
50mV
100ms
+150pA
50mV
100ms
+150pA
D.
10mV
100ms
-150pA

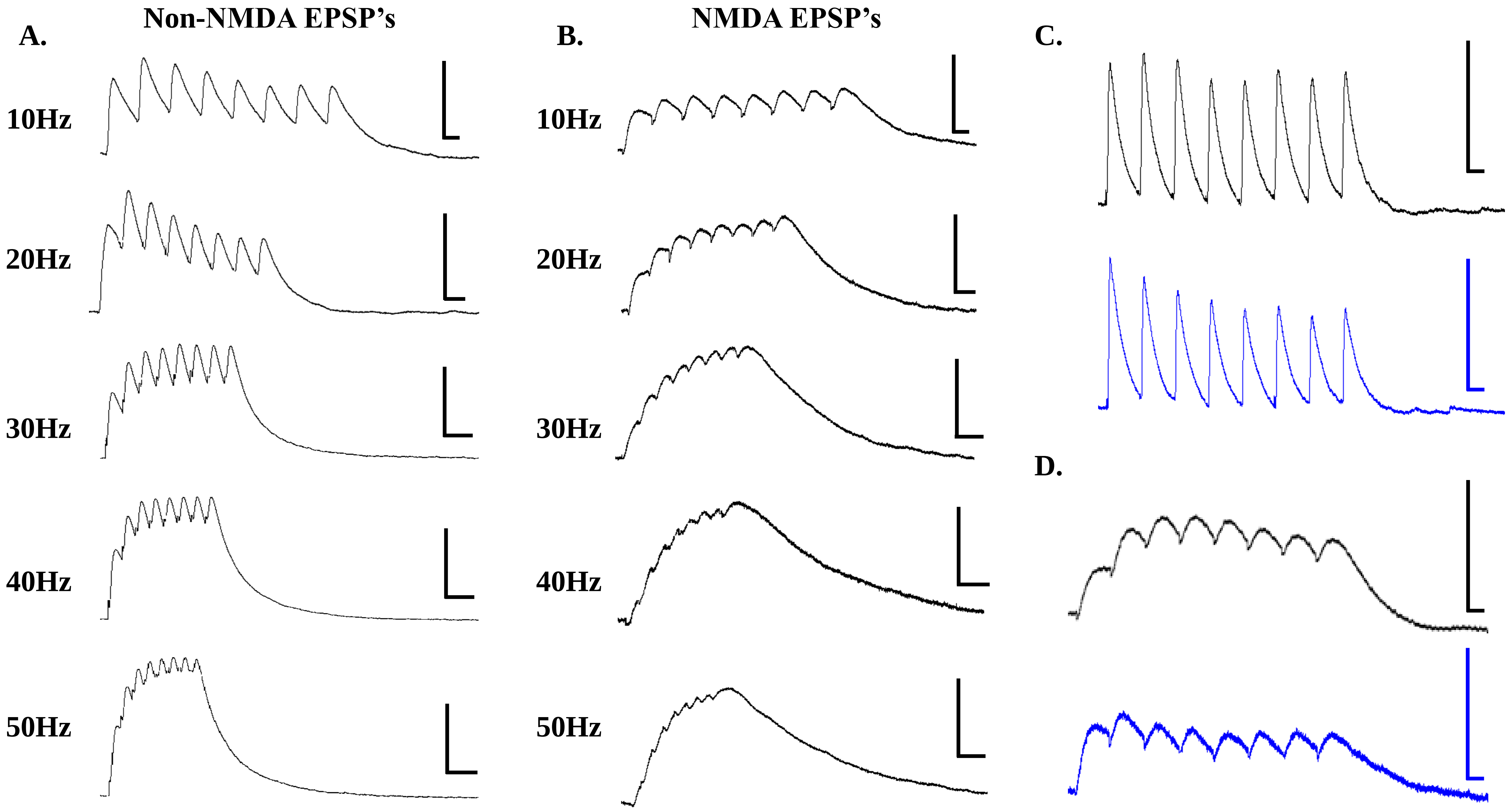

A.
Non-NMDA EPSP's
B.
NMDA EPSP's
C.
D.
10Hz
20Hz
30Hz
40Hz
50Hz
10Hz
20Hz
30Hz
40Hz
50Hz

A.

AMPA Layer V

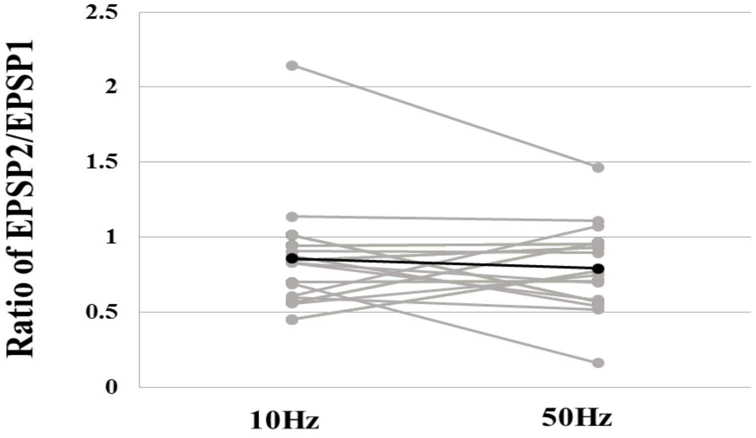

B.

AMPA Layer I

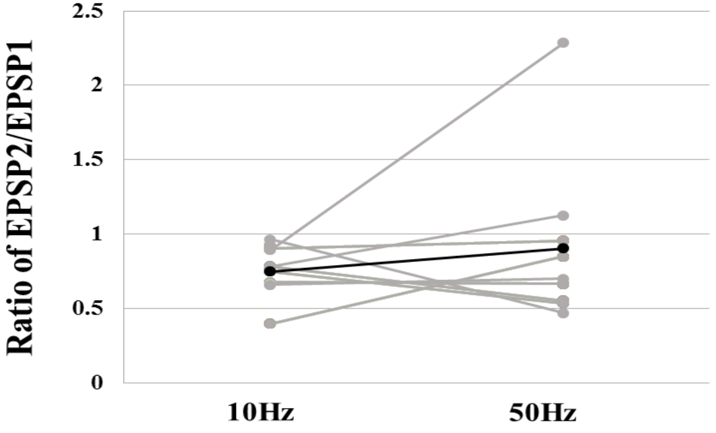

	Max Amplitude from baseline (mV±SEM)	Time to peak amplitude (ms±SEM)
10Hz Stimulation	9.8 ± 1.0 *	240 ± 40*
50Hz Stimulation	20.9 ± 2.0 *	102 ± 6*

	Max Amplitude from baseline (mV±SEM)	Time to peak amplitude (ms±SEM)
10Hz Stimulation	9.4 ± 0.9*	280 ± 60
50Hz Stimulation	22.2 ± 1.8*	100 ± 12

C.

NMDA Layer V

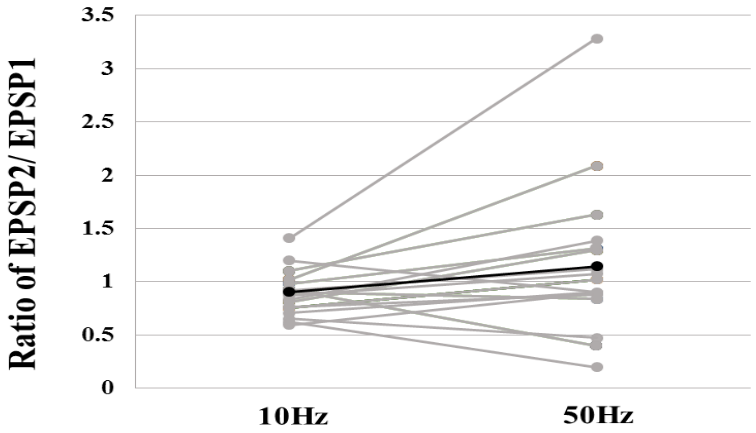

D.

NMDA Layer I

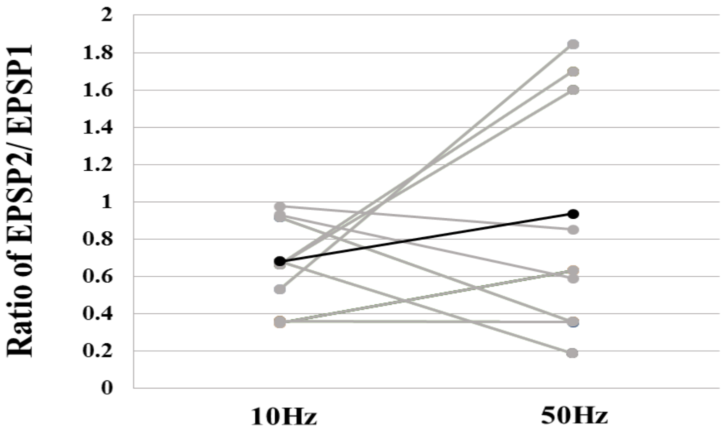

	Max Amplitude from baseline (mV±SEM)	Time to peak amplitude (ms±SEM)
10Hz Stimulation	4.5 ± 0.7*	420 ± 60*
50Hz Stimulation	11.2 ± 1.4*	154 ± 6*

	Max Amplitude from baseline (mV±SEM)	Time to peak amplitude (ms±SEM)
10Hz Stimulation	5.6 ± 1.0*	270 ± 70*
50Hz Stimulation	9.5 ± 1.5*	144 ± 14*

A.

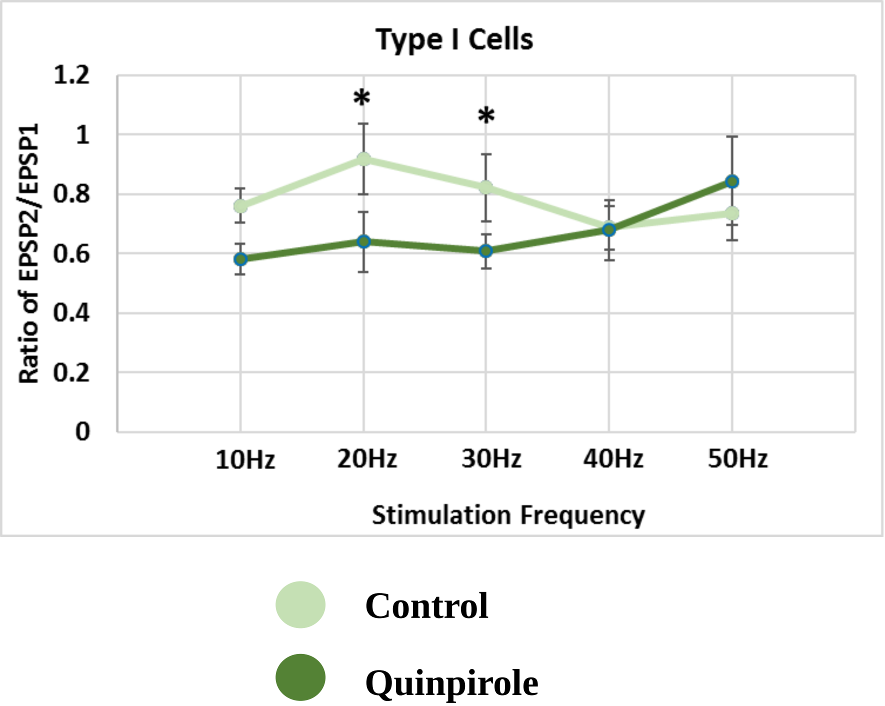

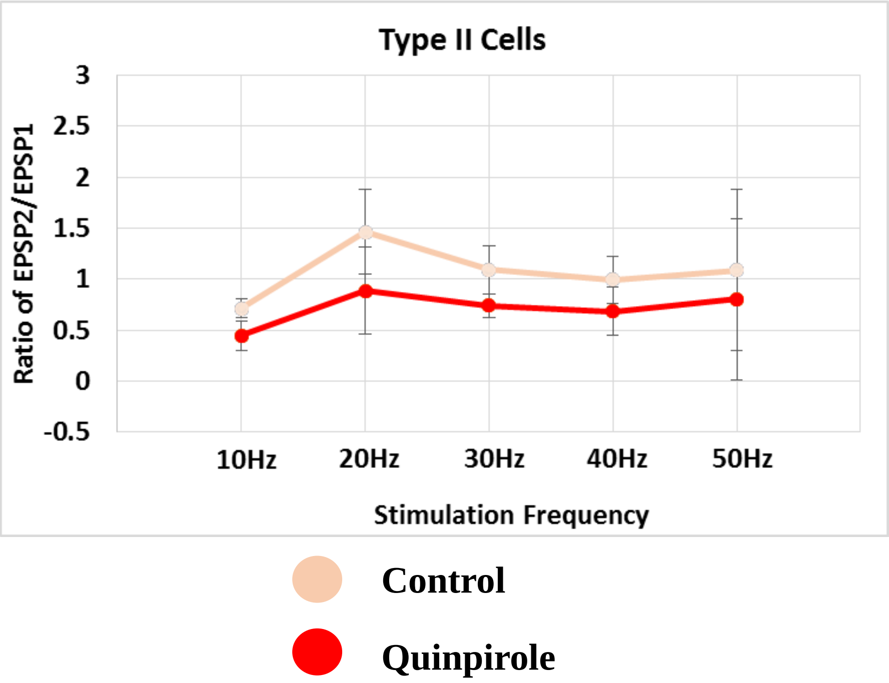

B.

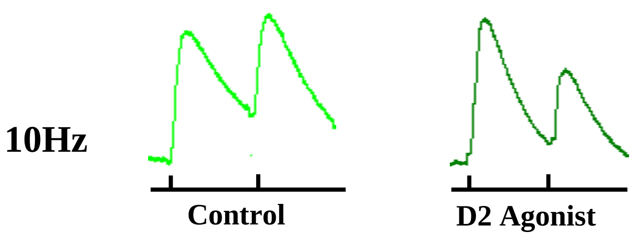

	EPSP1	EPSP2
Control	7.7±0.8 mV	5.9±0.7 mV
D2 Agonist	8.8±1.7 mV	4.8±0.6 mV

C.

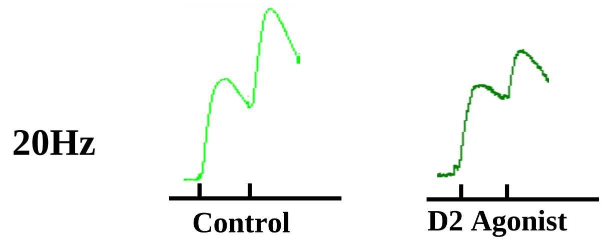

	EPSP1	EPSP2
Control	9.6±1.3 mV	8.7±1.8 mV
D2 Agonist	7.15±1.1 mV	4.3±0.6 mV

D.

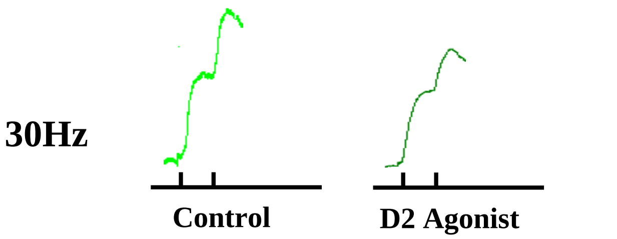

	EPSP1	EPSP2
Control	8.4±1.2 mV	6.8±1.1 mV
D2 Agonist	6.6±1.1 mV	3.9±0.6 mV

E.

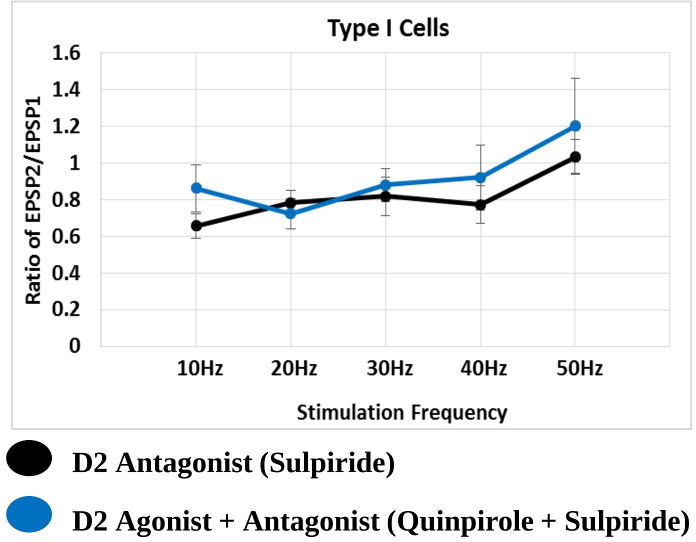

F.

	10Hz Stimulation		50Hz Stimulation	
	Max Amplitude from baseline (mV±SEM)	Time to peak amplitude (mV±SEM)	Max Amplitude from baseline (mV±SEM)	Time to peak amplitude (ms±SEM)
Control	9.4 ± 0.9	280 ± 60	22.2 ± 1.8	100 ± 12
D2 agonist	9.6 ± 1.5	130 ± 20	21.7 ± 4.0	106 ± 14

A.

B.

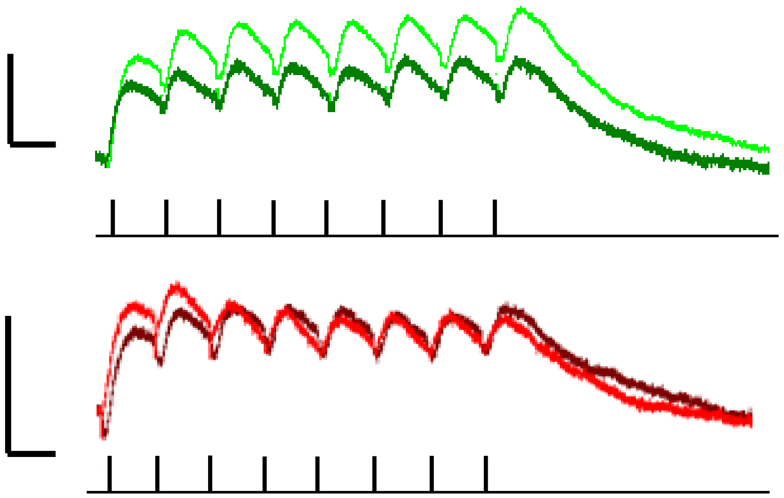

C.

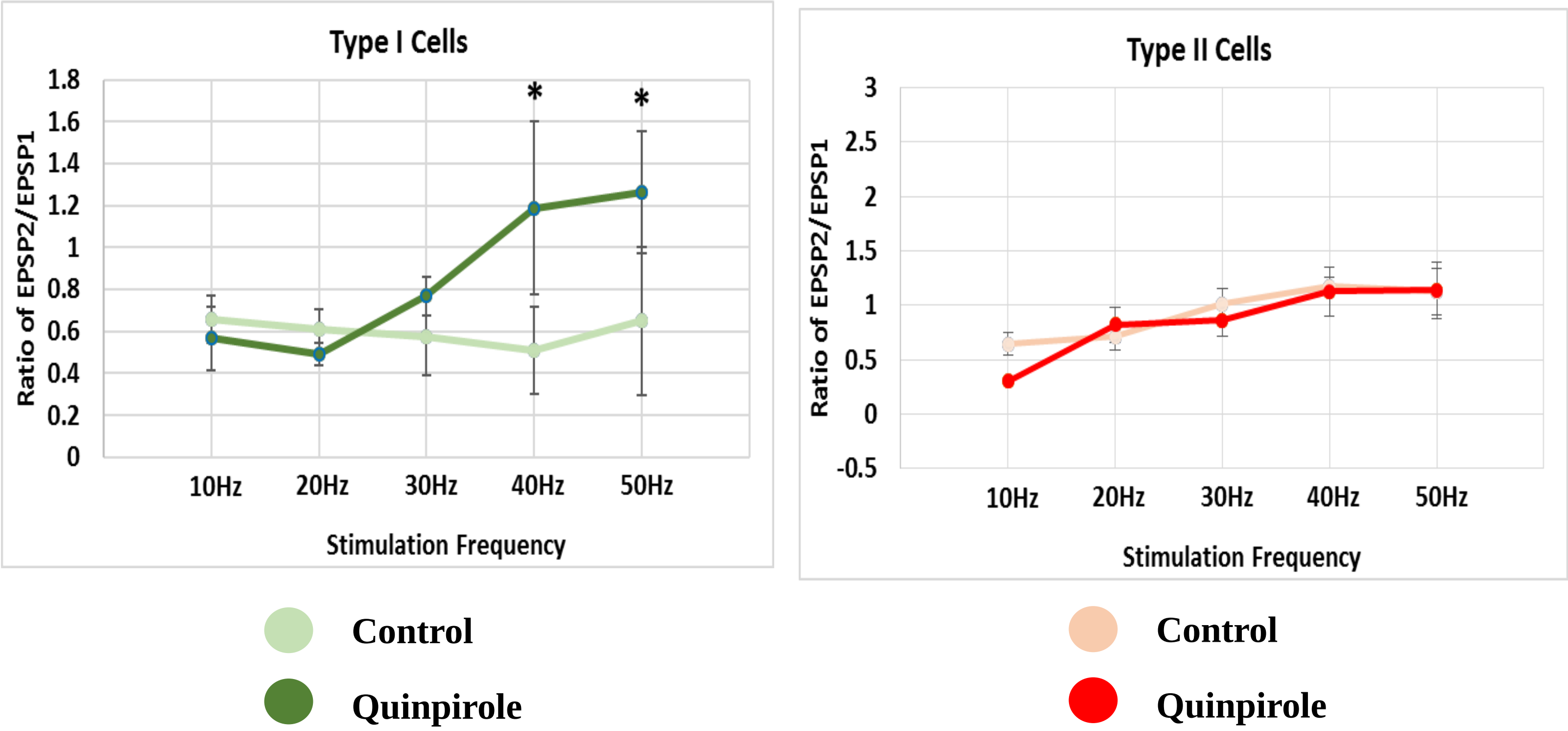

D.

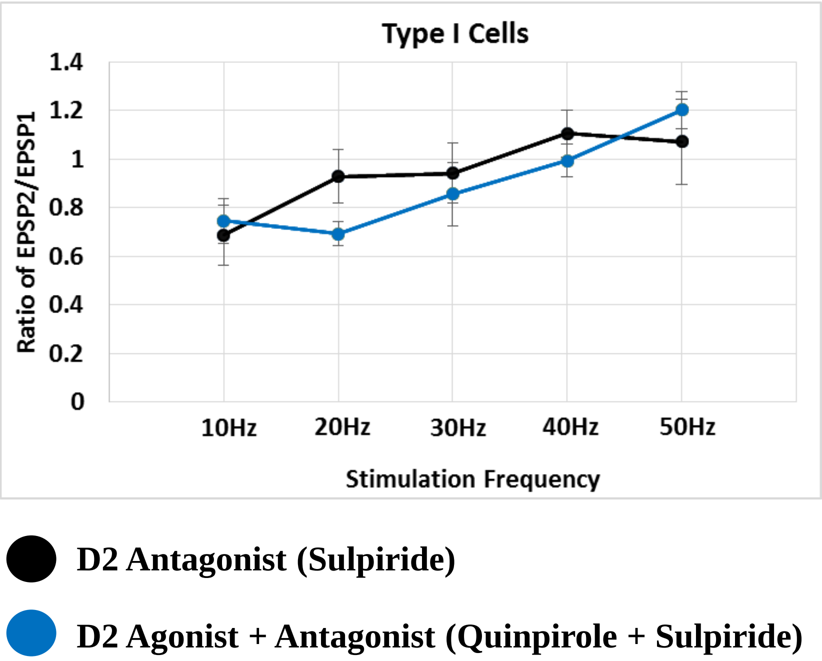

E.

	10Hz Stimulation		50Hz Stimulation	
	Max Amplitude from baseline (mV±SEM)	Time to peak amplitude (ms±SEM)	Max Amplitude from baseline (mV±SEM)	Time to peak amplitude (ms±SEM)
Control	5.6 ± 1.0	270 ± 70	9.5 ± 1.5	144 ± 14
D2 agonist	3.3 ± 0.5	430 ± 70	7.6 ± 1.5	160 ± 14
